# Supplementary material for: Asymmetric histone inheritance regulates olfactory stem cell fates during regeneration
Source: Nat Commun. 2026 Mar 23;17:4361. doi: 10.1038/s41467-026-70987-y (PMC13172531; doi:10.1038/s41467-026-70987-y)
Supplement: Supplementary file 2 — Reporting Summary [file 41467_2026_70987_MOESM2_ESM.pdf]

nature portfolio

Corresponding author(s): Xin Chen, Haiqing Zhao

Last updated by author(s): February 17, 2026

Reporting Summary

Nature Portfolio wishes to improve the reproducibility of the work that we publish. This form provides structure for consistency and transparency in reporting. For further information on Nature Portfolio policies, see our Editorial Policies and the Editorial Policy Checklist.

Statistics

For all statistical analyses, confirm that the following items are present in the figure legend, table legend, main text, or Methods section.

n/a	Confirmed	
☐	☒	The exact sample size (*n*) for each experimental group/condition, given as a discrete number and unit of measurement
☐	☒	A statement on whether measurements were taken from distinct samples or whether the same sample was measured repeatedly
☐	☒	The statistical test(s) used AND whether they are one- or two-sided<br>*Only common tests should be described solely by name; describe more complex techniques in the Methods section.*
☒	☐	A description of all covariates tested
☐	☒	A description of any assumptions or corrections, such as tests of normality and adjustment for multiple comparisons
☐	☒	A full description of the statistical parameters including central tendency (e.g. means) or other basic estimates (e.g. regression coefficient) AND variation (e.g. standard deviation) or associated estimates of uncertainty (e.g. confidence intervals)
☐	☒	For null hypothesis testing, the test statistic (e.g. F, t, r) with confidence intervals, effect sizes, degrees of freedom and P value noted<br>*Give P values as exact values whenever suitable.*
☒	☐	For Bayesian analysis, information on the choice of priors and Markov chain Monte Carlo settings
☐	☒	For hierarchical and complex designs, identification of the appropriate level for tests and full reporting of outcomes
☐	☒	Estimates of effect sizes (e.g. Cohen's *d*, Pearson's *r*), indicating how they were calculated

Our web collection on statistics for biologists contains articles on many of the points above.

Software and code

Policy information about availability of computer code

Data collection	Imaging Acquisition: Samples were imaged under a Leica SPE confocal microscope or STELLARIS 5 confocal microscope equipped with a Leica HC PL APO 63× 1.40-NA oil CS2 objective, a white light laser and power HyD S detector. Z-stacks of 0.5 µm per layer were taken for mitotic HBCs. The ImageJ software was used to quantify the fluorescent intensities and evaluate the division angle of anaphase and telophase HBCs.<br>Western Blot: The immunoreactive protein bands were visualized using a G:Box Chemi XRQ gel doc system (Syngene).
Data analysis	Co-localization Analysis: The co-localization assay was performed using FIJI (ImageJ) software. The image was imported into ImageJ, and the HBCs identified via P63 fluorescent signals. Drawing near the edge of the cell to the best of our ability, we outlined the HBCs of interest using the "freehand selections" tool on the toolbar. After outlining the cell, the selected area was duplicated using a function under the toolbar (Image>Duplicate Image). The duplicated area is then split into individual channels using the toolbar (Image>Color>Split Channels). With this, the images are ready for the co-localization analysis using the Coloc 2 plugin (Analysis>Co-localization>Coloc 2). The Coloc 2 tool implements and performs the pixel intensity correlation over space (pixel intensity spatial correlation analysis). After opening the Coloc 2 plugin pop-up, select the two desired channels to perform the analysis on into "Channel 1" and "Channel 2." We used Spearman's Rank Correlation value to compare co-localization between different datasets. The result is +1 for perfect correlation, 0 for no correlation, and -1 for perfect anti-correlation.<br>Single Cell RNA-seq and Data Analysis: The single cell RNA-seq (scRNA-seq) experiment was done following the G&T-seq protocol47. Single daughter cells after division of ex vivo cultured primary HBC cells were collected individually using mouth pipette with pairing information noted. 1 mL of pre-diluted (1:106) ERCC spike-in (Invitrogen Cat# 4456740) was added to the single cell lysate. RNA in the lysate was captured by oligo-dT beads and subjected to reverse transcription. cDNA from a single cell was amplified by 18 PCR cycles before dual indexing with the Nextera XT kit (Illumina Cat# FC-131-1096). The quality of single cell libraries was confirmed by TapeStation and libraries of 96 cells from 48 HBC pairs were pooled together for sequencing (150 bp paired-end) in one lane on the Illumina NovaSeq X Plus platform (Novogene US).<br>The quality of all FASTQ files from Illumina sequencing was analyzed and confirmed by FastQC (v0.12.1). scRNA-seq reads were trimmed with

Trimmomatic (v0.39)62. The trimmed RNA-seq reads with both pair mates were aligned by STAR (v2.7.11a)63 to the GRCm38 annotation (ENSEMBL release 100) plus ERCC information. StringTie (v2.2.1)64 was used to generate counts of genes in the GTF reference. After aligning all the RNA-seq reads of the 96 (48 pairs) single cells to the reference genome, the total number of different genes detected in each single cell was examined and only the cells that captured more than 10,000 genes would be kept for downstream analysis. Meanwhile, the cells that have more than 5% of the reads aligned to mitochondrial genes would be eliminated from downstream analysis. All 96 cells met the quality control criteria and were retained for downstream analysis.

Publicly available single-cell RNA-seq datasets of in vivo retrieved HBC cells were downloaded from Gene Expression Omnibus (accession numbers: GSE99251, GSE95601)12,46. scRNA-seq count matrices of all single cells were analyzed by Seurat (v5.1.0)65. The "CCAIntegration" method in Seurat was used when integrating the published scRNA-seq data into the newly generated HBC pair datasets before visualization in UMAP. The newly generated scRNA-seq dataset in this study was deposited to Gene Expression Omnibus (accession numbers: GSE286046).

After clustering of the single cells from HBC pairs by Seurat, the RNA-seq results of all cells falling into the "Activated HBC" cluster were treated as one group and compared to data of cells from the "Renewed HBC" cluster using DESeq2 (v1.44.0)66. ERCC spike-in was used for normalization. Log2 fold change = ±1 with adjusted R value < 0.1 was used as cutoff for significantly up- or down-regulated genes. Significantly upregulated genes were then used for gene ontology analysis with DAVID67. ERCC normalized gene counts were used for violin plots, and R values were calculated by a two-tailed t test.

Statistical Analysis: Statistical evaluation was performed by Mann-Whitney unpaired t-test and Chi-square test. Data are presented as Average ± SEM and significant difference between two groups were noted by asterisks: * $p < 0.05$, ** $p < 0.01$, *** $p < 0.001$, **** $p < 0.0001$. Statistical analysis was performed by GraphPad Prism 10 software. The fluorescence intensity was measured by Image J software.

For manuscripts utilizing custom algorithms or software that are central to the research but not yet described in published literature, software must be made available to editors and reviewers. We strongly encourage code deposition in a community repository (e.g. GitHub). See the Nature Portfolio guidelines for submitting code & software for further information.

Data

Policy information about availability of data

All manuscripts must include a data availability statement. This statement should provide the following information, where applicable:

- Accession codes, unique identifiers, or web links for publicly available datasets
- A description of any restrictions on data availability
- For clinical datasets or third party data, please ensure that the statement adheres to our policy

The scRNA-seq dataset in this study was deposited to Gene Expression Omnibus (GSE286046).

Research involving human participants, their data, or biological material

Policy information about studies with human participants or human data. See also policy information about sex, gender (identity/presentation), and sexual orientation and race, ethnicity and racism.

Reporting on sex and gender	N/A
Reporting on race, ethnicity, or other socially relevant groupings	N/A
Population characteristics	N/A
Recruitment	N/A
Ethics oversight	N/A

Note that full information on the approval of the study protocol must also be provided in the manuscript.

Field-specific reporting

Please select the one below that is the best fit for your research. If you are not sure, read the appropriate sections before making your selection.

☒ Life sciences ☐ Behavioural & social sciences ☐ Ecological, evolutionary & environmental sciences

For a reference copy of the document with all sections, see nature.com/documents/nr-reporting-summary-flat.pdf

Life sciences study design

All studies must disclose on these points even when the disclosure is negative.

Sample size	At least 3 mice were chosen as the biological replicates for the experiments including tissue cryosection and staining.<br>Due to the inherent variability of behavioral tests, 16 control and 19 NZ-treated animals were used for the buried food seeking behavior test on Day 15 post-MMZ injection; 7 control and 9 NZ-treated animals were used for the buried food seeking behavior test on Day 28 post-MMZ injection. Similarly, 8 control and 10 NZ-treated animals were used for the olfactory preference test on Day 15 and Day 28 post-MMZ injection.
Data exclusions	No data exclusions except the serial dilution results of Western blot (clarified in the Figure 1F legend). Due to the low signals of 1:4 dilution lane, the first two lanes were used for quantification. H4-mScarlet amount is ~6% of the endogenous H4.

Replication	All experiments were repeated at least 3 times independently to assure reproducibility. All attempts for replication shows similar results.
Randomization	For each experiment, both control and treatment groups were randomly selected.
Blinding	Sample preparation, data collection and analysis were performed blindly.

Reporting for specific materials, systems and methods

We require information from authors about some types of materials, experimental systems and methods used in many studies. Here, indicate whether each material, system or method listed is relevant to your study. If you are not sure if a list item applies to your research, read the appropriate section before selecting a response.

Materials & experimental systems

n/a	Involved in the study	
☐	☒	Antibodies
☒	☐	Eukaryotic cell lines
☒	☐	Palaeontology and archaeology
☐	☒	Animals and other organisms
☒	☐	Clinical data
☒	☐	Dual use research of concern
☒	☐	Plants

Methods

n/a	Involved in the study	
☒	☐	ChIP-seq
☒	☐	Flow cytometry
☒	☐	MRI-based neuroimaging

Antibodies

Antibodies used	The primary antibodies and dilutions used were as follows: mouse monoclonal anti-p63, 1:100 (D-9, sc-25268; Santa Cruz); rabbit monoclonal anti-Ki67, 1:250 (NB600-1252, NOVUS); rabbit polyclonal anti-RNA polymerase II RPB1 phospho S2, 1:800 (AB5095, Abcam); rabbit polyclonal anti-RNA polymerase II RPB1 phospho S5, 1:800 (AB5131, Abcam); rat monoclonal anti-RNA polymerase II CTD, 1:200 (61082, Active Motif); rabbit monoclonal anti-Histone H4, 1:200 (Ab10158, Abcam), rabbit monoclonal anti-Histone H3, 1:200 (A17562, ABclonal); rabbit monoclonal anti-Histone H3.3, 1:250 (NBP2-67530, NOVUS); ChromoTek Histone-Label Atto488 (for H2A-H2B), 1:400 (tba488, Proteintech); rabbit monoclonal anti-phospho-Histone H3Thr3 (JY325), 1:800 (05-746R, Millipore Sigma); Goat anti-Olfactory Marker Protein (OMP), 1:500 (544-10001, WAKO). For Western blot, anti-H4 antibodies (1:10,000, Sigma, 05-858) was used.
Validation	All the used antibodies have been validated by the Research Resource Identifiers (#RRID) and the manufacturer with publications.<br>Validation: p63 (RRID): AB_628092; Ulibarri, Max R., et al. "Epithelial organoid supports resident memory CD8 T cell differentiation." Cell reports 43.8 (2024).<br>Ki67 (RRID): AB_2142376; van Schaik, Tom, et al. "Dynamic chromosomal interactions and control of heterochromatin positioning by Ki-67." EMBO reports 23.12 (2022): e55782.<br>RNA Pol IIS2ph (RRID): AB_304749; Wang, Zihao, et al. "Dcr1 senses R-loops for RNAPII termination at sites of replication stress and repair pathway choice." Molecular Cell 85.21 (2025): 3947-3964.<br>RNA Pol IIS5ph (RRID): AB_449369; Stein, Chad B., et al. "Integrator endonuclease drives promoter-proximal termination at all RNA polymerase II-transcribed loci." Molecular cell 82.22 (2022): 4232-4245.<br>RNA Pol II CTD (RRID): AB_2793500; Cai, Guodi, et al. "UMP functions as an endogenous regulator of NR4A1 to control gastric cancer progression." Molecular Cell 85.23 (2025): 4347-4364.<br>H4 (RRID): AB_296888; Warner, James L., et al. "The histone chaperone Spt6 controls chromatin structure through its conserved N-terminal domain." Molecular Cell 85.18 (2025): 3407-3424.<br>H3 (RRID): AB_2770395; Fu, Yu, et al. "The low-dose CHK1 inhibitor prexasertib triggers VDAC1 dephosphorylation to activate mtDNA-STING signaling and synergize immunotherapy." Cell Reports 44.5 (2025).<br>H3.3 (RRID): AB_3353601; https://www.novusbio.com/products/histone-h33-antibody-st50-08_nbp2-67530?srsltid=AfmBOor5y2XNjSRZL_99EX7ITnJu8P0D6h6x-0CrCVkNBrr3u4RMhs71#reviews-publications<br>H2A-H2B (RRID): AB_2827588; Tanaka, Nobuyuki, et al. "Three-dimensional single-cell imaging for the analysis of RNA and protein expression in intact tumour biopsies." Nature Biomedical Engineering 4.9 (2020): 875-888.<br>H3T3p (RRID): AB_10863137; Evano, Brendan, et al. "Dynamics of asymmetric and symmetric divisions of muscle stem cells in vivo and on artificial niches." Cell reports 30.10 (2020): 3195-3206.<br>OMP (RRID): AB_664696; Yang, Lu M., Sung-Ho Huh, and David M. Ornitz. "FGF20-expressing, Wnt-responsive olfactory epithelial progenitors regulate underlying turbinate growth to optimize surface area." Developmental cell 46.5 (2018): 564-580.<br>H4 (RRID): AB_390138; Bellelli, Roberto, et al. "POLE3-POLE4 is a histone H3-H4 chaperone that maintains chromatin integrity during DNA replication." Molecular cell 72.1 (2018): 112-126.

Animals and other research organisms

Policy information about studies involving animals; ARRIVE guidelines recommended for reporting animal research, and Sex and Gender in Research

Laboratory animals	All experiments were performed on adult mice at 6-8 weeks of age. The mice were maintained with open access to food and water

under a 12-hour light/12-hour dark cycle. TRE3G-H4-mScarlet and TRE3G-H3.3-mScarlet mouse strains were obtained from Sue Hammoud laboratory, p63-EGFP reporter mouse strain was from Sinha laboratory, and B6N.FVB(Cg)-Tg(CAG-rtTA3)4288Slowe/J mouse strain was form Jackson lab (Strain #:016532).

Wild animals	N/A
Reporting on sex	Both male and female animals were used in all experiments.
Field-collected samples	N/A
Ethics oversight	All animal experiments were conducted according to the procedures approved by the Johns Hopkins University IACUC guidelines (approval protocol numbers MO19A127/MO22A71/MO24A114).

Note that full information on the approval of the study protocol must also be provided in the manuscript.

Plants

Seed stocks	N/A
Novel plant genotypes	N/A
Authentication	N/A
